# miR-137: A New Player in Schizophrenia

**DOI:** 10.3390/ijms15023262

**Published:** 2014-02-21

**Authors:** Jingwen Yin, Juda Lin, Xudong Luo, Yanyan Chen, Zheng Li, Guoda Ma, Keshen Li

**Affiliations:** 1 Institute of Neurology, Guangdong Medical College, Zhanjiang 524001, China; E-Mails: amysays@live.com (J.Y.); xiaohonghuacyy@163.com (Y.C.); 2 Department of Psychiatry, Affiliated Hospital of Guangdong Medical College, Zhanjiang 524001, China; E-Mails: linjuda@126.com (J.L.); lxd708@163.com (X.L.); 3 Unit on Synapse Development and Plasticity, National Institute of Mental Health, National Institutes of Health, Bethesda, MD 20892, USA; E-Mail: lizheng2@mail.nih.gov

**Keywords:** miR-137, schizophrenia, neurodevelopment, target gene, signaling pathway

## Abstract

Schizophrenia is a complex genetic disease and characterized by affective, cognitive, neuromorphological, and molecular abnormalities that may have a neurodevelopmental origin. MicroRNAs (miRNAs) are critical to neurodevelopment and adult neuronal processes by modulating the activity of multiple genes within biological networks. MiR-137 as a brain-enriched microRNA, plays important roles in regulating embryonic neural stem cells (NSCs) fate determination, neuronal proliferation and differentiation, and synaptic maturation. Its dysregulation causes changes in the gene expression regulation network of the nervous system, thus inducing mental disorders. Recently, miR-137 has been confirmed as a gene related to schizophrenia susceptibility. In the following review, we summarize the expression pattern, epigenetic regulation and functions of miR-137. A more complete picture of the miR-137, which is dysregulated in psychiatric illness, may improve our understanding of the molecular mechanisms underlying schizophrenia.

## 1. Introduction

Schizophrenia is a disease with phenotypic heterogeneity that involves multiple candidate genes[1]. It is characterized by nervous system dysfunction caused by the “accumulation” of dysregulation events in susceptibility genes [2]. In the nervous system, the ability of miRNAs to have cascade effects on gene expression and functional pathways precisely regulate many processes, including embryonic and adult neurogenesis, synaptic development, dendritic protein synthesis, axon guidance, and neural plasticity. The dysregulation of miRNA expression causes abnormalities in neural structure and function [3–5] and results in neurodevelopmental aspects of psychiatric disorders [6]. Autopsy results revealed that compared with those in normal individuals, the expression levels of several miRNAs changed in regions of the brain, such as the prefrontal cortex, the thalamus, and the temporal lobe in schizophrenia patients [7,8]. This result further confirmed that miRNAs might participate in the pathogenesis of schizophrenia.

MiR-137 is a miRNA that is highly expressed in the brain [9]. It can regulate both neurogenesis and synaptic plasticity, and recently it has been confirmed as an important susceptibility-related gene for schizophrenia. The purpose of this paper was to summarize the regulatory function of miR-137 in neurodevelopment and schizophrenia as well as to improve our understanding of the molecular mechanisms underlying schizophrenia.

## 2. Expression and Epigenetic Regulation of miR-137

MiR-137 is expressed in neural stem cells (NSCs) and many regions of the brain, including the amygdala, hippocampus, orbital frontal cortex, nucleus accumbens, anterior cingulate cortex, dorsolateral prefrontal cortex, caudate nucleus, putamen, and thalamus [9,10]. The highest miR-137 expression levels were observed in amygdala and the hippocampus [9], especially in adult brain region with active neurogenesis, the subgranular cell layer in the hippocampal dentate gyrus [10]. Autopsy results on schizophrenia patients indicated that the miR-137 expression level in the dorsolateral prefrontal cortex was significantly decreased compared to that in the control group [9]. This result also suggested a relationship between miR-137 and schizophrenia.

MiR-137 is located on chromosome 1p21.3. There is a large number of CpG islands in the upstream 2.5 kb promoter region of this gene [11], suggesting that miR-137 expression may be affected by the methylation level of its promoter. Studies have shown that miR-137 expression has the following epigenetic regulatory mechanisms.

**Figure 1.**
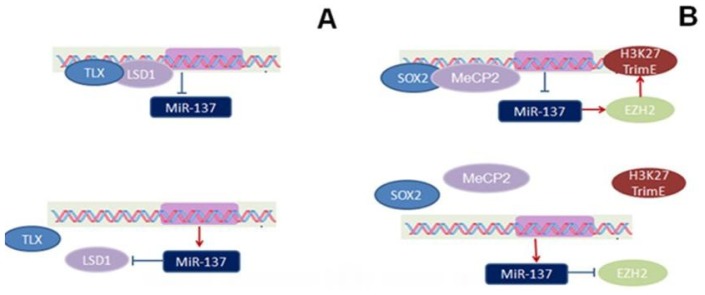
Expression regulation of miR-137. (**A**) In embryonic NSCs, TLX represses the expression of miR-137 by recruiting LSD1 to the genomic regions of miR-137. On the other hand, miR-137 suppresses LSD1 expression to inhibit its binding to TLX. Therefore, miR-137 forms a negative feedback regulatory loop with TLX and LSD1 to ensure its expression is maintained at an appropriate level; (**B**) In aNSCs, MeCP2 along with Sox2 mediates the epigenetic regulation of miR-137. One target of miR-137, Ezh2, is a histone H3 lysine 27 methyltransferase. The miR-137–mediated repression of Ezh2 feeds back to chromatin, resulting in a global decrease in histone H3 trimethyl lysine 27.

In embryonic NSCs, miR-137 expression is regulated by the nuclear receptor transcription factor TLX and the histone demethylase LSD1 (lysine specific demethylase 1). TLX recruits its corepressor LSD1 to form a complex and then binds to a genomic region of miR-137 gene to inhibit the transcription of pre-miR-137. Remarkably, LSD1 is a target gene of miR-137, the inhibition of LSD1 by miR-137 promotes miR-137 expression accordingly. Therefore, miR-137 forms a negative feedback regulatory loop with LSD1 and TLX to ensure its expression is maintained at an appropriate level [12] (Figure 1A). TLX plays an important role in regulating cell cycle progression in NSCs of the developing brain, in part through transcriptional repression of its downstream target genes. The expression of TLX and LSD1 is relatively high in NSCs and decreased upon differentiation. Inversely, miR-137 expression was induced in differentiated cells. Theoretically, the expression of miR-137 could be repressed by increased TLX and LSD1, thus maintaining NSCs in an undifferentiated and self-renewable state [10,12,13] (Figure 1A).

In adult neural stem cells (aNSCs), the regulatory system for miR-137 expression consists of the methyl CpG binding protein 2 (MeCP2), the transcription factor Sox2, and the Histone methyltransferase enhancer of zeste homolog 2 (Ezh2) [11]. MeCP2 is a key member of the methyl CpG binding protein family and is an important transcriptional repressor [14]. MeCP2 interacts with the transcription factor Sox2 to form a complex that binds to the promoter region of miR-137 and establishes an epigenetic state in the chromatin to inhibit miR-137 transcription. In addition, the miR-137-mediated repression of Ezh2 can feedback to chromatin in the form of a global decrease in histone H3 trimiethyl lysine (H3-K27-Tri-Me). Coexpression of Ezh2 can rescue phenotypes associated with miR-137 overexpression, suggesting another regulatory loop in epigenetic regulation of miR-137 [11,15] (Figure 1B). Therefore, the crosstalk between miR-137 and epigenetic regulation may coordinately regulate aNSCs fate.

## 3. The Function of miR-137 in the Nervous System

MiR-137 regulates the dynamics between proliferation and differentiation during different neural development stages. In the NSCs, miR-137 overexpression can induce cell cycle arrest to start differentiation [13,15]. Therefore, it has both proliferation inhibition and differentiation promotion functions. MiR-137 blocks the transition from the G1 phase to the S phase mainly through the inhibition of cyclin-dependent kinases (such as CDK6) to cause cell cycle arrest of NSCs and promote their entry into the differentiation stage [12,13,15–17]. The possible role of miR-137 in the promotion of cell differentiation is also supported by the observations in the other stem cells derived from oligodendroglioma or glioblastoma multiforme [13]. Interestingly, in a separate study, miR-137 has distinctly opposite effects on aNSCs, where increased expression of miR-137 promotes proliferation and inhibits differentiation [11]. In addition, an increased CDK6 protein was observed in miR-137-overexpressing aNSCs [11]. These results suggest that an essential and context-dependent function of miR-137 during multiple neurogenesis developmental stages.

After differentiation, miR-137 has been implicated in neuronal maturation, including dendritic morphogenesis, phenotypic maturation, and spine development both in brain and cultured primary neurons. Smrt *et al*. [10] discovered that miR-137 can inhibit the transcription of Mind bomb 1 (Mib-1) through the conserved target site located in the 3′ untranslated region of Mib1 mRNA. Mib-1 is a ubiquitin ligase located on postsynaptic membranes. It can regulate the synaptic lengths of neuronal cells. MiR-137 targets Mib-1 to regulate neuronal maturation and dendritic morphogenesis, thus affecting the structure and function of neurons.

In addition, miR-137 participates in the regulation of signal transduction between synapses and long-term potentiation (LTP) through the regulation of another target gene, cyclooxygenase-2 (COX-2). In the nervous system, COX-2 is selectively expressed in dendrites and dendritic spines of excitatory neurons in the cortex, hippocampus, and amygdala [18]. A metabolite of COX-2, prostaglandin E2 (PGE2), can bind to presynaptic membrane EP_2/4_ receptors to activate the cAMP/PKA signaling pathway, thus activating calcium-dependent protein kinases, increasing glutamate release from hippocampal synapses, and maintaining neural signal transduction [19]. As a retrograde messenger, COX-2 promotes the release of presynaptic membrane neurotransmitters [20]. Therefore, we assumed that miR-137 might regulate neuronal excitability, synaptic plasticity, and signal transmission between nerve cells through the inhibition of COX-2.

## 4. Neural Development- and Schizophrenia-Related Downstream Target Genes and Signaling Pathways Regulated by miR-137

Neuronal maturation and communication leads to the establishment of functional neural circuits that mediate sensory and motor processing, and underlie behavior. Abnormalities in neural structure and function can cause brain function disorders, including schizophrenia [21]. The regulation of neurodevelopment by miR-137 may be an important cause of schizophrenia. In a genome-wide association study, Ripke *et al*. [22] showed that miR-137 was the gene with the strongest association with schizophrenia; they also proposed four other predicted miR-137 target genes that were associated with schizophrenia. These genes included transcription factor 4 (TCF-4); a calcium channel, voltage-dependent, L type, alpha1C subunit gene (CACNA1C); CSMD1; and C10orf26. Subsequently, these genes have been confirmed to be miR-137 target genes [23].

TCF-4 is a nuclear transcription factor and an important regulatory factor for a sensory gating channel [24]. Excessive, irrelevant stimuli caused by sensory gating impairment might underlie cognitive and attention disorders in schizophrenia patients. Mice with TCF-4 overexpression showed schizophrenia-related symptoms, and schizophrenia patients had reduced TCF-4 expression in the prefrontal cortex, which was negatively correlated with miR-137 [9]. These results indicated that abnormal TCF-4 expression may cause psychotic symptoms.

CACNA1C encodes a calcium channel, voltage-dependent, L type, 1C subunit. Many genome-wide association studies showed that it was associated with mental diseases such as schizophrenia, bipolar disorder, depressive disorders, and autism [25–27]. The 1C subunit is coupled with cell membrane depolarization, which increases the permeability of the membrane to calciumions, thus resulting in the activation of intracellular signaling pathways. The impairment of this process might cause abnormal information processing and results in mental diseases such as schizophrenia [27].

Furthermore, zinc finger protein 804A (ZNF804A) is another miR-137 target gene that has been confirmed to be closely associated with schizophrenia [28]. ZNF804A functions in the regulation of neuronal migration and synaptic plasticity. It participates in dopamine signaling pathways, and its target gene, catechol-*O*-methyltransferase (COMT), can directly and selectively degrade dopamine in prefrontal cortex synapses through methylation [29]. When COMT expression is inhibited, the reduced dopamine degradation in prefrontal cortex synapses causes dopamine hyperactivity, which might result in schizophrenia. The other ZNF804A target gene associated with the dopamine pathway is the dopamine receptor D2 (DRD2) gene [29]. When the dopaminergic system in the mesolimbic pathway is hyperactive, the hyperactivation of the postsynaptic membrane D2 receptor can induce positive symptoms, such as hallucinations and delusions [30]. Antipsychotic drugs include mainly DRD2 blockers, which can attenuate abnormal excitation to inhibit symptoms of schizophrenia.

Wright *et al*. [31] performed an analysis on biological pathways associated with predicted miR-137 target genes. These target genes formed a regulatory network coordinating neural structure and function. The three major pathways were the LTP, ephrin (Eph) receptor, and axonal guidance signaling pathways. Axon guidance allows nerve cell axons to reach target regions along the correct path [32] and permits contact between neuronal synapses to connect their functions. LTP affects the efficiency of information transmission between synapses [33]. The Eph receptor and its ligand not only participate in axon pathfinding but also regulate the formation of neural networks, the formation of the neural tube and paraxial mesoderm, and cell migration guidance [34,35]. The impairment of these basic structures that support brain function or abnormalities in information transmission result in reduced information processing efficiency, thus causing abnormalities in specific brain regions and resulting in symptoms of schizophrenia. For example, schizophrenia patients have excessively strong reactions to neutral stimuli and demonstrate impairment of emotion recognition; this phenomenon may result in delusions.

**Figure 2.**
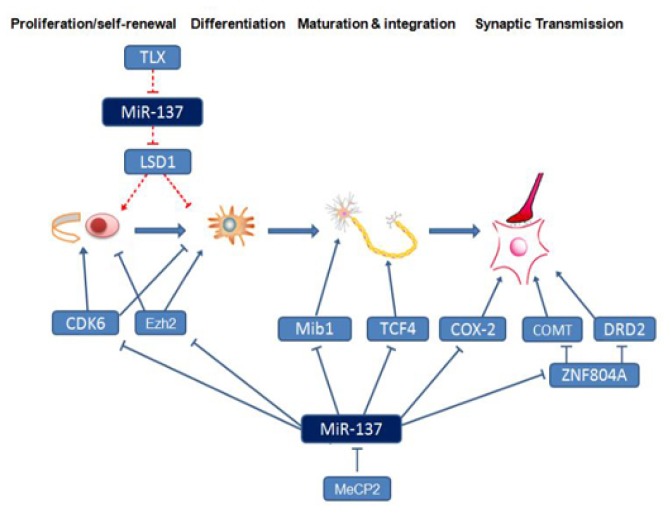
The role of gene regulatory networks of miR-137 in neurogenesis and maturation. Inhibitory effect found in embryos: 
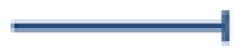
, stimulatory effect found in embryos: 
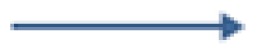
, Inhibitory effect found in adults: 
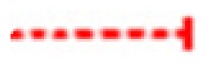
, stimulatory effect found in adults: 
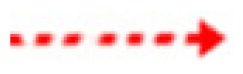
.

Therefore, as an upstream regulatory factor, miR-137 may influence the pathogenesis of schizophrenia by regulating neurodevelopment and schizophrenia-related genes through the regulatory network (Figure 2).

## 5. Correlations between miR-137 Single Nucleotide Polymorphisms and Schizophrenia

Guan *et al*. [36] and Egawa *et al*. [37] studied the association between many single nucleotide polymorphism (SNP) loci of miR-137 (rs4634961, rs9324387, rs2391905, rs4950117, rs1938568, rs4950101, rs2802535, rs2660304, g.98511534 G > C, g.98511769 G > T, and g.98511780 T > C) and schizophrenia. Except for the rs1625579 genotype and the corresponding gene frequency, which showed a significant correlation with Chinese Han people with schizophrenia compared to the control group, the genotypes did not correlate with schizophrenia.

Rs1625579 is located at an 8 kb region downstream of the miR-137 gene. It is clearly the locus that has the strongest correlation with schizophrenia (*p* = 1.6 × 10^−11^) [22]. Correlation studies on populations in the UK [38,39], Australia [40], China [36], the USA, and Canada [41] showed that this locus affected many phenotypes of schizophrenia (Table 1). Lett *et al*. [41] showed that in the North American population, the age of onset of patients with the TT genotype at that location was significantly earlier than that of people carrying the G allele (*p* = 3.1 × 10^−5^). Imaging examinations showed that the TT genotype patients had reduced white matter density in the brain, diminished hippocampal volume, and enlarged lateral ventricles [41]. These phenotypes may result from the abnormal development of neural structure and the late maturation of functions caused by the regulation of neural growth by miR-137. van Erp *et al*. [42] showed that patients with the rs1625579 TT genotype had dorsolateral prefrontal cortex hyperactivation on working memory, indicating that patients with the TT genotype had lower prefrontal cortex neural performance on working memory. This symptom may result in cognitive dysfunction in schizophrenia patients. The study of Green *et al*. [40] used a series of cognitive tests to examine cognitive function in schizophrenia patients. These tests included the Letter Number Sequencing Test to measure working memory and the Controlled Oral Word Association Test to assess executive functioning. Based on the scores of these tests, the patients were divided into the cognitive deficit (CD) group and the cognitively spared (CS) group. In the CD group, the polymorphism rs1625579 was associated with negative symptoms. The studies of Cummings *et al*. [38] on mental disease patients (such as those suffering from schizophrenia, schizoaffective disorder, and bipolar affective disorder) showed that patients with the T allele had lower mental symptom scores and more cognitive deficits. Therefore, the rs1625579 polymorphisms in miR-137 may affect many phenotypes related to schizophrenia.

## 6. Summary and Prospects

The role of miR-137 in schizophrenia by has gradually become a focus. The target genes regulated by miR-137 participate in several aspects of structural development and functional maturation of the brain. Functional studies on these target genes preliminarily described the basic outline of the gene regulation network in schizophrenia, and miR-137 plays a central role in this network. However, currently, direct evidence for the overexpression/knockout of miR-137 *in vivo* is still lacking. Wright *et al*. [31] used a bioinformatic method to predict that there were 1,144 potential miR-137 target genes. Of these, 25 genes were schizophrenia susceptibility-related genes. So far, there are few confirmed downstream target genes, indicating that our understanding of the molecular functions of miR-137 is still not comprehensive. Further validation and functional studies on potential target genes of miR-137 will provide an overall systematic understanding of miR-137 functions in schizophrenia and may provide new targets for the development of new drugs for schizophrenia based on the network level of regulation.

## Figures and Tables

**Table 1.** Summary of human genetic studies linking the miR-137 rs1625579 polymorphism to schizophrenia and endophenotypes.

Population	Number of participants		Genotype frequencies (%)			*p*-value	Correlated phenotypes	*p*-value
			TT	GT	GG			
American, Canadian (Lett *et al*.)	510	SZ	67.45	29.8	2.75	>0.05 *	age-at-onset and white matter integrity	3.1 × 10^−5^ *
	121	CTR	62.81	33.06	4.13			3.88 × 10^−8^ *
Australian (Green *et al*.)	491	SZ	64.97	31.16	3.87	0.85 *	cognitive deficits in combination with greater severity of negative symptoms	0.017 *
	328	CTR	62.5	33.23	4.27			
Scottish (Whalley *et al*.)	44	SZ	59.09	38.64	2.27	>0.05	activation in the posterior right medial frontal gyrus, BA 6	<0.001
	81	CTR	61.73	35.8	2.47			
American (Theo *et al*.)	48	SZ	81.25	18.75	0	0.47 *	left DLPFC activation	0.02 *
	63	CTR	73.02	23.81	3.17			
Chinese (Guan *et al*.)	1430	SZ	77.7	20.8	1.5	0.023		
	1570	CTR	74	23.6	2.4			

Abbreviations: SZ, schizophrenia cases; CTR, healthy controls; BA, Brodmann area; DLPFC, Dorsal Lateral Prefrontal Cortex;

* Statistical analyses compared TT with GT/GG genotypes.

## References

[1] Allen N.C., Bagade S., McQueen M.B., Ioannidis J.P., Kavvoura F.K., Khoury M.J., Tanzi R.E., Bertram L. (2008). Systematic meta-analyses and field synopsis of genetic association studies in schizophrenia: The SzGene database. Nat. Genet.

[2] Cannon T.D. (1996). Abnormalities of brain structure and function in schizophrenia: implications for aetiology and pathophysiology. Ann. Med.

[3] Murchison E.P., Partridge J.F., Tam O.H., Cheloufi S., Hannon G.J. (2005). Characterization of Dicer-deficient murine embryonic stem cells. Proc. Natl. Acad. Sci. USA.

[4] Wang Y., Medvid R., Melton C., Jaenisch R., Blelloch R. (2007). DGCR8 is essential for microRNA biogenesis and silencing of embryonic stem cell self-renewal. Nat. Genet.

[5] Stark K.L., Xu B., Bagchi A., Lai W.S., Liu H., Hsu R., Wan X., Pavlidis P., Mills A.A., Karayiorgou M. (2008). Altered brain microRNA biogenesis contributes to phenotypic deficits in a 22q11-deletion mouse model. Nat. Genet.

[6] Sewell R.A., Perry E.B., Karper L.P., Bell M.D., Lysaker P., Goulet J.L., Brenner L., Erdos J., d’Souza D.C., Seibyl J.P. (2010). Clinical significance of neurological soft signs in schizophrenia: Factor analysis of the Neurological Evaluation Scale. Schizophr. Res.

[7] Perkins D.O., Jeffries C.D., Jarskog L.F., Thomson J.M., Woods K., Newman M.A., Parker J.S., Jin J., Hammond S.M. (2007). microRNA expression in the prefrontal cortex of individuals with schizophrenia and schizoaffective disorder. Genome Biol.

[8] Xu B., Karayiorgou M., Gogos J.A. (2010). MicroRNAs in psychiatric and neurodevelopmental disorders. Brain Res.

[9] Guella I., Sequeira A., Rollins B., Morgan L., Torri F., van Erp T.G., Myers R.M., Barchas J.D., Schatzberg A.F., Watson S.J. (2013). Analysis of miR-137 expression and rs1625579 in dorsolateral prefrontal cortex. J. Psychiatr. Res.

[10] Smrt R.D., Szulwach K.E., Pfeiffer R.L., Li X., Guo W., Pathania M., Teng Z.Q., Luo Y., Peng J., Bordey A. (2010). MicroRNA miR-137 regulates neuronal maturation by targeting ubiquitin ligase mind bomb-1. Stem Cells.

[11] Kunej T., Godnic I., Horvat S., Zorc M., Calin G.A. (2012). Cross talk between microRNA and coding cancer genes. Cancer J.

[12] Sun G., Ye P., Murai K., Lang M.F., Li S., Zhang H., Li W., Fu C., Yin J., Wang A. (2011). miR-137 forms a regulatory loop with nuclear receptor TLX and LSD1 in neural stem cells. Nat. Commun.

[13] Silber J., Lim D.A., Petritsch C., Persson A.I., Maunakea A.K., Yu M., Vandenberg S.R., Ginzinger D.G., James C.D., Costello J.F. (2008). miR-124 and miR-137 inhibit proliferation of glioblastoma multiforme cells and induce differentiation of brain tumor stem cells. BMC Med.

[14] Lewis J.D., Meehan R.R., Henzel W.J., Maurer-Fogy I., Jeppesen P., Klein F., Bird A. (1992). Purification, sequence, and cellular localization of a novel chromosomal protein that binds to methylated DNA. Cell.

[15] Szulwach K.E., Li X., Smrt R.D., Li Y., Luo Y., Lin L., Santistevan N.J., Li W., Zhao X., Jin P. (2010). Cross talk between microRNA and epigenetic regulation in adult neurogenesis. J. Cell Biol.

[16] Jiang K., Ren C., Nair V.D. (2013). MicroRNA-137 represses Klf4 and Tbx3 during differentiation of mouse embryonic stem cells. Stem Cell Res.

[17] Tarantino C., Paolella G., Cozzuto L., Minopoli G., Pastore L., Parisi S., Russo T. (2010). miRNA 34a, 100, and 137 modulate differentiation of mouse embryonic stem cells. FASEB J.

[18] Breder C.D., Dewitt D., Kraig R.P. (1995). Characterization of inducible cyclooxygenase in rat brain. J. Comp. Neurol.

[19] Akaneya Y. (2007). The remarkable mechanism of prostaglandin E2 on synaptic plasticity. Gene Regul. Syst. Biol.

[20] Sang N., Zhang J., Marcheselli V., Bazan N.G., Chen C. (2005). Postsynaptically synthesized prostaglandin E2 (PGE2) modulates hippocampal synaptic transmission via a presynaptic PGE2 EP2 receptor. J. Neurosci.

[21] Guilmatre A., Dubourg C., Mosca A.L., Legallic S., Goldenberg A., Drouin-Garraud V., Layet V., Rosier A., Briault S., Bonnet-Brilhault F. (2009). Recurrent rearrangements in synaptic and neurodevelopmental genes and shared biologic pathways in schizophrenia, autism, and mental retardation. Arch. Gen. Psychiatry.

[22] Ripke S., Sanders A., Kendler K., Levinson D., Sklar P., Holmans P. (2011). Genome-wide association study identifies five new schizophrenia loci. Nat. Genet.

[23] Kwon E., Wang W., Tsai L.H. (2011). Validation of schizophrenia-associated genes CSMD1, C10orf26, CACNA1C and TCF4 as miR-137 targets. Mol. Psychiatry.

[24] Brzozka M.M., Radyushkin K., Wichert S.P., Ehrenreich H., Rossner M.J. (2010). Cognitive and sensorimotor gating impairments in transgenic mice overexpressing the schizophrenia susceptibility gene Tcf4 in the brain. Biol. Psychiatry.

[25] Green E.K., Grozeva D., Jones I., Jones L., Kirov G., Caesar S., Gordon-Smith K., Fraser C., Forty L., Russell E. (2010). The bipolar disorder risk allele at CACNA1C also confers risk of recurrent major depression and of schizophrenia. Mol. Psychiatry.

[26] Dao D.T., Mahon P.B., Cai X., Kovacsics C.E., Blackwell R.A., Arad M., Shi J., Zandi P.P., O’Donnell P., Knowles J.A. (2010). Mood disorder susceptibility gene CACNA1C modifies mood-related behaviors in mice and interacts with sex to influence behavior in mice and diagnosis in humans. Biol. Psychiatry.

[27] Bhat S., Dao D.T., Terrillion C.E., Arad M., Smith R.J., Soldatov N.M., Gould T.D. (2012). CACNA1C (Cav1.2) in the pathophysiology of psychiatric disease. Prog. Neurobiol.

[28] Kim A.H., Parker E.K., Williamson V., McMichael G.O., Fanous A.H., Vladimirov V.I. (2012). Experimental validation of candidate schizophrenia gene ZNF804A as target for hsa-miR-137. Schizophr. Res.

[29] Girgenti M.J., LoTurco J.J., Maher B.J. (2012). ZNF804a regulates expression of the schizophrenia-associated genes PRSS16, COMT, PDE4B, and DRD2. PLoS One.

[30] Liu L., Yuan G., Cheng Z., Zhang G., Liu X., Zhang H. (2013). Identification of the mRNA expression status of the dopamine d2 receptor and dopamine transporter in peripheral blood lymphocytes of schizophrenia patients. PLoS One.

[31] Wright C., Turner J.A., Calhoun V.D., Perrone-Bizzozero N. (2013). Potential impact of miR-137 and its targets in schizophrenia. Front. Genet.

[32] Skaper S.D. (2012). Neuronal growth-promoting and inhibitory cues in neuroprotection and neuroregeneration. Methods Mol. Biol.

[33] Yang Y., Calakos N. (2013). Presynaptic long-term plasticity. Front. Synaptic Neurosci.

[34] Du J., Fu C., Sretavan D.W. (2007). Eph/ephrin signaling as a potential therapeutic target after central nervous system injury. Curr. Pharm. Des.

[35] Nikolov D.B., Xu K., Himanen J.P. (2013). Eph/ephrin recognition and the role of Eph/ephrin clusters in signaling initiation. Biochim. Biophys. Acta.

[36] Guan F., Zhang B., Yan T., Li L., Liu F., Li T., Feng Z., Liu X., Li S. (2014). MIR137 gene and target gene CACNA1C of miR-137 contribute to schizophrenia susceptibility in Han Chinese. Schizophr. Res.

[37] Arinami T., Ohtsuki T., Ishiguro H., Ujike H., Tanaka Y., Morita Y., Mineta M., Takeichi M., Yamada S., Imamura A. (2005). Genomewide high-density SNP linkage analysis of 236 Japanese families supports the existence of schizophrenia susceptibility loci on chromosomes 1p, 14q, and 20p. Am. J. Hum. Genet.

[38] Cummings E., Donohoe G., Hargreaves A., Moore S., Fahey C., Dinan T.G., McDonald C., O’Callaghan E., O’Neill F.A., Waddington J.L. (2013). Mood congruent psychotic symptoms and specific cognitive deficits in carriers of the novel schizophrenia risk variant at MIR-137. Neurosci. Lett.

[39] Whalley H.C., Papmeyer M., Romaniuk L., Sprooten E., Johnstone E.C., Hall J., Lawrie S.M., Evans K.L., Blumberg H.P., Sussmann J.E. (2012). Impact of a microRNA MIR137 Susceptibility Variant on Brain Function in People at High Genetic Risk of Schizophrenia or Bipolar Disorder. Neuropsychopharmacology.

[40] Green M.J., Cairns M.J., Wu J., Dragovic M., Jablensky A., Tooney P.A., Scott R.J., Carr V.J. (2013). Genome-wide supported variant MIR137 and severe negative symptoms predict membership of an impaired cognitive subtype of schizophrenia. Mol. Psychiatry.

[41] Lett T.A., Chakravarty M.M., Felsky D., Brandl E.J., Tiwari A.K., Goncalves V.F., Rajji T.K., Daskalakis Z.J., Meltzer H.Y., Lieberman J.A. (2013). The genome-wide supported microRNA-137 variant predicts phenotypic heterogeneity within schizophrenia. Mol. Psychiatry.

[42] Van Erp T.G., Guella I., Vawter M.P., Turner J., Brown G.G., McCarthy G., Greve D.N., Glover G.H., Calhoun V.D., Lim K.O. (2014). Schizophrenia miR-137 locus risk genotype is associated with dorsolateral prefrontal cortex hyperactivation. Biol. Psychiatry.

